# Reduced CTGF Expression Promotes Cell Growth, Migration, and Invasion in Nasopharyngeal Carcinoma

**DOI:** 10.1371/journal.pone.0064976

**Published:** 2013-06-03

**Authors:** Yan Zhen, Yanfen Ye, Xiaoli Yu, Chunping Mai, Ying Zhou, Yan Chen, Huiling Yang, Xiaoming Lyu, Ye Song, Qiangyun Wu, Qiaofen Fu, Mengyang Zhao, Shengni Hua, Hao Wang, Zhen Liu, Yajie Zhang, Weiyi Fang

**Affiliations:** 1 Cancer Research Institute of Basic Medicine School, Southern Medical University, Guangzhou, PR China; 2 Department of Pathology of Basic School, Medical University of Guangzhou, Guangzhou, PR China; 3 School of Pharmacy, Guangdong Medical College, Dongguan, PR China; 4 Department of Neurosurgery, Nanfang Hospital, Southern Medical University, Guangzhou, PR China; Thomas Jefferson University, United States of America

## Abstract

**Background:**

The role of CTGF varies in different types of cancer. The purpose of this study is to investigate the involvement of CTGF in tumor progression and prognosis of human nasopharyngeal carcinoma (NPC).

**Experimental design:**

CTGF expression levels were examined in NPC tissues and cells, nasopharynx (NP) tissues, and NP69 cells. The effects and molecular mechanisms of CTGF expression on cell proliferation, migration, invasion, and cell cycle were also explored.

**Results:**

NPC cells exhibited decreased mRNA expression of CTGF compared to immortalized human nasopharyngeal epithelial cell line NP69. Similarly, CTGF was observed to be downregulated in NPC compared to normal tissues at mRNA and protein levels. Furthermore, reduced CTGF was negatively associated with the progression of NPC. Knocking down CTGF expression enhanced the colony formation, cell migration, invasion, and G1/S cell cycle transition. Mechanistic analysis revealed that CTGF suppression activated FAK/PI3K/AKT and its downstream signals regulating the cell cycle, epithelial-mesenchymal transition (EMT) and MMPs. Finally, DNA methylation microarray revealed a lack of hypermethylation at the CTGF promoter, suggesting other mechanisms are associated with suppression of CTGF in NPC.

**Conclusion:**

Our study demonstrates that reduced expression of CTGF promoted cell proliferation, migration, invasion and cell cycle progression through FAK/PI3K/AKT, EMT and MMP pathways in NPC.

## Introduction

CTGF is a cysteine-rich, matrix-associated, heparin-binding protein, and is widely expressed in variety human tissues and organs, such as connective tissue, pancreas, placenta, and lung. Its expression has been associated with tumor cell proliferation, adhesion, and angiogenesis [1], [2] and serves as a prognostic marker in many types of human cancer [3]–[5]. Interestingly, CTGF plays different roles in different types of cancer. In pancreatic cancer, prostate cancer, liver cancer, breast cancer, and sarcoma, CTGF has been shown to be an oncogenic factor promoting tumor progression [1], [6]–[9]. Conversely, CTGF functions as a tumor suppressor in lung cancer, ovarian cancer, and oral squamous cell cancer [5], [10], [11]. The expression pattern and functional mechanisms of CTGF in NPC have not been established.

Nasopharyngeal carcinoma (NPC) is a tumor arising from the epithelial cells that cover the surface and line the nasopharynx. Its highest incidence worldwide occurs in Southern China, with an age-standardized incidence rate varying from 20 to 50 cases per 100,000 people. Typical cervical lymph node metastases frequently occur in early stages. Synergetic effects of viral infections, genetic alterations, and environmental factors are thought to drive abnormal gene expression, which contributes to the initiation and development of NPC [12]–[15]. In a previous study, cDNA microarray was utilized to examine differentially expressed genes between NPC tissues and non-cancerous nasopharyngeal tissues. Through BRB-array tool analysis, the expression of connective tissue growth factor (CTGF), a member of CCN family, was found to be notably downregulated in NPC tissues, suggesting a potential role in suppressing the pathogenesis of NPC [12].

In order to further clarify the role of CTGF in the pathogenesis of NPC, we investigated its expression and correlation with clinicopathologic features in NPC patients, as well as its effects on cell growth, cell cycle, migration, and invasion in cell lines. Our studies demonstrated that reduced CTGF expression stimulates cell proliferation, migration, invasion and cell cycle progression via FAK/PI3K/AKT signaling, EMT and MMP pathways.

## Materials and Methods

### Cell Culture and Sample Collection

Eight NPC cell lines 5–8F, 6–10B, CNE2, CNE1, C666–1, HONE1, HNE1 and SUNE1 were obtained from Cancer Research Institute of Southern Medical University. All cell lines were maintained in RPMI 1640 medium supplemented with 10% newborn calf serum (NBCS) (PAA Laboratories, Inc, Pasching, Austria). NP69, an immortalized human nasopharyngeal epithelial cell line, was grown in defined-KSFM medium supplemented with epidermal growth factor (EGF) (Invitrogen, Carlsbad, USA). All cell lines were incubated in a humidified chamber with 5% CO_2_ at 37°C. 20 fresh primary NPC tissues, 11 fresh NP tissuses, 92 paraffin-embedded undifferentiated primary NPC specimens and 25 paraffin-embedded NP specimens were obtained at the time of diagnosis before any therapy from People’s Hospital in Zhongshan City (Guangdong, China). The clinical processes were approval from the Ethics Committees of People’s Hospital of Zhongshan City and patients provided informed consent. The pathologic stage of all specimens was confirmed according to the 1997 NPC staging system of the UICC.

### Reanalysis of Microarray Data

Microarray data set (GEO accession number: GSE2370) between five representative EBV-negative NPC cell lines: TW01, TW03, TW04, TW06 and CGBM1 and normal nasal mucosal epithelial submitted by Lee was retrieved from the GEO database and differentially expressed genes were screened with a selection criteria based on our previous investigation [12].

### RNA Isolation, Reverse Transcription, and qRT-PCR

RNA was extracted from the NPC cell lines, NP69 cell line, NPC tissues and NP tissues using Trizol(Takara, Shiga, Japan). For CTGF, RNA was transcribed into cDNA and amplified with specific sense: 5′CTGCCCTCGCGGCTTAC3′; antisense primer: 5′TGGTGCAGCCAGAAAGCTCA3′. The assays were performed in accordance with manufacturer’s instructions (Takara, Shiga, Japan). The PCR reaction for each gene was repeated three times. ARF5 was used as internal control [13].

### Immunohistochemistry and Evaluation of Staining

Immunohistochemistry and evaluation of staining of CTGF (Santa Cruz Biotechnology, Santa Cruz, USA) were performed in NPC and noncancerous nasopharyngeal tissues according to a previous description [14].

### Western Blot Analysis

Western blot was carried out according as described [13] with rabbit polyclonal anti-CTGF antibody (1∶200; Epitomics, Burlingame, USA), anti-PCNA antibody (1∶1000; Epitomics, Burlingame, USA), anti-ACTB,CCND1,CDK4, p21, E2F1, and p15 antibody (1∶400; Santa Cruz Biotechnology, Santa Cruz, USA), anti-pRb(Ser,780), MMP2, MMP9, FAK, pFAK(Tyr397), AKT, pAKT(Ser473), PI3K, pPI3K(Tyr458), Snail, E-Cadherin, N-Cadherin and Vimentin antibody(1∶1000; Cell signaling technology, Danvers, USA). An HRP-conjugated anti-rabbit IgG antibody was used as the secondary antibody (Zhongshan, Beijing, China). Signals were detected using enhanced chemiluminescence reagents (Pierce, Rockford, IL).

### Establishment of NPC 6–10B Cell Line with Stable Expression of CTGF Short Hairpin RNA

The preparation of lentivirus expressing human CTGF short hairpin RNA (shRNA-1024,1047) (Table 1) was performed using the pLVTHM-GFP lentiviral RNAi expression system [16]. NPC 6–10B cells were infected with lentiviral particles containing specific or negative control vectors, and the polyclonal cells with GFP signals were selected for further experiments using FACS flow cytometry.

**Table 1:** shRNA sequences for CTGF.

CTGF		Sequence
1024	Sense	5′CGCGTCCCCGCACCAGCATGAAGACATACCTTCAAGAGAGGTATGTCTTCATGCTGGTGCTTTTTGGAAAT 3′
	Antisense	5′CGATTTCCAAAAAGCACCAGCATGAAGACATACCTCTCTTGAAGGTATGTCTTCATGCTGGTGCGGGGA 3′
1047	Sense	5′CGCGTCCCCGCTAAATTCTGTGGAGTATGTTTCAAGAGAACATACTCCACAGAATTTAGCTTTTTGGAAAT 3′
	Antisense	5′CGATTTCCAAAAAGCTAAATTCTGTGGAGTATGTTCTCTTGAAACATACTCCACAGAATTTAGCGGGGA 3′

### Transient Transfection with siRNAs

Small-interfering RNA (siRNA) for CTGF was designed and synthesized by Guangzhou RiboBio (RiboBio Inc, China). The sequence of each gene and their controls are shown in Table 2. Twenty-four hours prior to transfection, NPC cells 6–10B and HONE1 were plated onto a 6-well plate or a 96-well plate (Nest, Biotech,China) at 30–50% confluence. They were then transfected into cells using TurboFect™ siRNA Transfection Reagent (Fermentas, Vilnius, Lithuania**)** according to the manufacturer's protocol. Cells were collected after 48–72 hr for the further experiments.

**Table 2:** SiRNA sequences of CTGF.

No		Sequence
1	Sense	5′GCACCAGCAUGAAGACAUA dTdT 3′
	Antisense	3′dTdT CGUGGUCGUACUUCUGUAU 5′
2	Sense	5′CCAGACCCAACUAUGAUUA dTdT 3′
	Antisense	3′ dTdT GGUCUGGGUUGAUACUAAU5′
3	Sense	5′GUGCAUCCGUACUCCCAAA dTdT 3′
	Antisense	3′dTdT CACGUAGGCAUGAGGGUUU5′

### CCK8 Assay

The rate of *in vitro* cell viability was assessed using CCK8 assay. Cells were seeded in 96-well plates at a density of 1,000 cells/well. For shRNA-CTGF, the cells were incubated for 1, 2, 3, 4, 5, 6, or 7 d. For siRNA-CTGF, the cells were incubated for 1, 2, or 3d. Ten microliters of CCK8 (Cell Counting Kit-8, Beyotime, China) was added to each well and incubated for 2 h. The absorbance value (OD) of each well was measured at 450 nm. For each experimental condition, 5 wells were used. Experiments were performed three times.

### Colony Formation Assay

Cells were plated in 6-well culture plates at 100 cells/well. Each cell group had 2 wells. After incubation for 12 days at 37°C, cells were washed twice with PBS and stained with Giemsa solution. The number of colonies containing ≥50 cells was counted under a microscope. The colony formation efficiency was calculated as (number of colonies/number of cells inoculated)×100%.

### Cell Cycle Analysis

Cell cycle examination was carried out according to previous description [14]. The DNA content of labeled cells was acquired using FACS cytometry assay(BD Biosciences).

### *In vitro* Cell Migration and Invasion Assays

*In vitro* cell migration and invasion assays were examined according to our previous study (13). Briefly, 1×10^5^ cells were seeded on a fibronectin-coated polycarbonate membrane insert in a transwell apparatus (Corning, Corning, USA). After the cells were incubated for 12 h, Giemsa-stained cells adhering to the lower surface were counted under a microscope in five predetermined fields (100×). For the cell invasion assay, the procedure was similar to the cell migration assay, except that the transwell membranes were pre-coated with 24 µg/µl Matrigel (R&D Systems, USA).

### Examination of CTGF Promoter Methylation by DNA Methylation Microarray Assay

The examination procedure for NimbleGen DNA methylation microarray for 17 NPCs and 3 NP tissues has been described [14], [17]. All experiments were performed at the Kangchen Biology Corporation, Shanghai, China.

### Statistical Analysis

All data were analyzed for statistical significance using SPSS 13.0 software. The unpaired T test was applied to test the differential mRNA expression of CTGF in NPC tissues compared to NP tissues. The Chi-square test was used to examine the differences of CTGF protein expression between normal epithelium and cancer tissues of nasopharynx. The Chi-square test was applied to the examination of relationship between CTGF expression levels and clinicopathologic characteristics. One-way ANOVA was used to determine the differences between groups *in vitro* analyses. A P value of less than 0.05 was considered statistically significant.

## Results

### CTGF is Expressed at Low Levels in NPC

**Figure 1:**
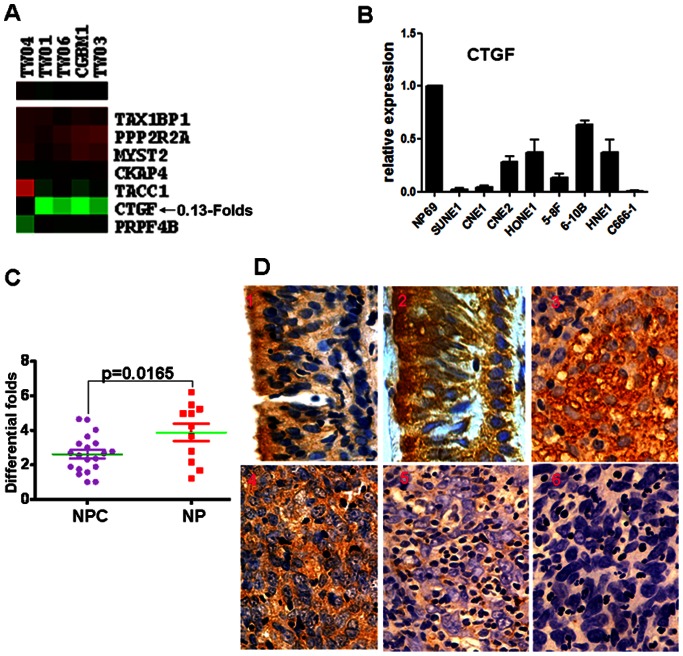
Reduced expression of CTGF promotes the progression and poor prognosis of NPC patients. **A.** Decreased CTGF expression was shown in NPC cells compared to nasopharynx tissues by microarray data analysis of GSE2370 set retrieved from the GEO database (Red color: high expression,Green color:low expression). **B.** Compared with immortalized human nasopharyngeal epithelial NP69 cell line, CTGF expression was significantly downregulated in 8 NPC cell lines. **C.** Compared with 11 NP tissues, CTGF expression was markedly decreased in 20 primary NPC tissues (p = 0.0165). The unpaired t test was used for this assay (*p<0.05). **D.** CTGF expression was decreased in 92 primary NPC samples compared to 25 NP tissues 1,2)High expression in nasopharyngeal epithelium; 3,4)High expression in NPC; 5,6): Low expression in NPC(Original magnification 400×). The Chi-square test was used for this assay (*p<0.05).

From Lee et al’s microarray data (GSE2370), CTGF was more weakly expressed in NPC tissues compared to NP tissues by an average of 0.13 fold (Figure 1A). This expression pattern was similar to our microarray data comparing NPC cells and NP tissues (12). QPCR analysis indicated that the CTGF expression level was significantly decreased in 8 NPC cell lines in comparison to the immortalized human nasopharyngeal epithelial cell lines NP69 (Figure 1B). Furthermore, downregulation of CTGF mRNA was also observed in NPC tissues compared to normal nasopharyngeal tissues(p = 0.0165) (Figure 1C). We also measured the expression levels of CTGF protein in archived paraffin-embedded NPC samples and NP specimens using immunohistochemical staining (Figure 1D). Specific CTGF protein staining was found in the cytoplasm of non-cancerous and malignant nasopharynx tissues. CTGF protein was highly expressed in NP epithelium compared to NPC samples (P<0.001) (Table 3).

**Table 3:** Downregulation of CTGF protein in NPC compared to NP epithelium tissues.

Group	Protein expression (n)			P Value
	Total	Low	High	
Normal epithelium	25	1	24	
Cancer	92	53	39	p<0.0001#

# Chi-square Test.

### Downregulated Protein Expression of CTGF is Negatively Associated with NPC Progression

We analyzed the relationship between clinicopathologic characteristics and CTGF expression levels in individuals with NPC (Table 4). We did not find a significant association of CTGF expression levels with patient's age, sex, smoking status, family tumor history, disease recurrence, or distant metastasis (M classification) in 92 NPC cases. However, we observed that the reduced expression level of CTGF was markedly correlated with tumor size (T classification) (P = 0.036), lymph node metastasis (N classification) (N0–N1 vs. N2–N3) (P = 0.020), and clinical stage (I–II vs. III–IV) (P = 0.027) in NPC patients.

**Table 4:** Correlation between the clinicopathologic characteristics and expression of CTGF protein in NPC.

Characteristics	n	CTGF (%)		*P*
		High expression	Low expression	
Gender				
Male	63	27(42.9)	36 (57.1)	
Female	29	16(55.2)	13 (44.8)	0.369
Age(y)				
≥50	40	18 (45.0)	22 (55.0)	
<50	52	25 (48.1)	27(51.9)	0.853
Smoking				
Yes	15	5 (33.3)	10 (66.7)	
No	77	36 (46.8)	41 (53.2)	0404
Family tumor history				
Yes	2	0(0.0)	2(100.0)	
No	90	40(44.4)	50(55.6)	0.503
Recurrence				
Yes	20	8(40.0)	12(60.0)	
No	72	29(40.3)	43(59.7)	1.000
T classification				
T_1_–T_2_	66	35(53.0)	31 (47.0)	
T_3_–T_4_	26	7 (26.9)	19(73.1)	0.036
N classification				
N_0_–N_1_	51	28 (54.9)	23(45.1)	
N_2_–N_3_	41	12(29.3)	29 (70.7)	0.020
Distant metastasis				
Yes	5	1(20.0)	4(80.0)	
No	87	41(47.1)	46(52.9)	0.371
TNM Clinical stage				
I∼II	32	20 (62.5)	12 (37.5)	
III∼IV	60	22(36.7)	38(63.3)	0.027

### Stably Downregulated or Transiently Inhibited CTGF Expression Stimulated Cell Proliferation, the Plate Clone Formation in vitro and Increased Cell Cycle Transition from G1 to S

**Figure 2:**
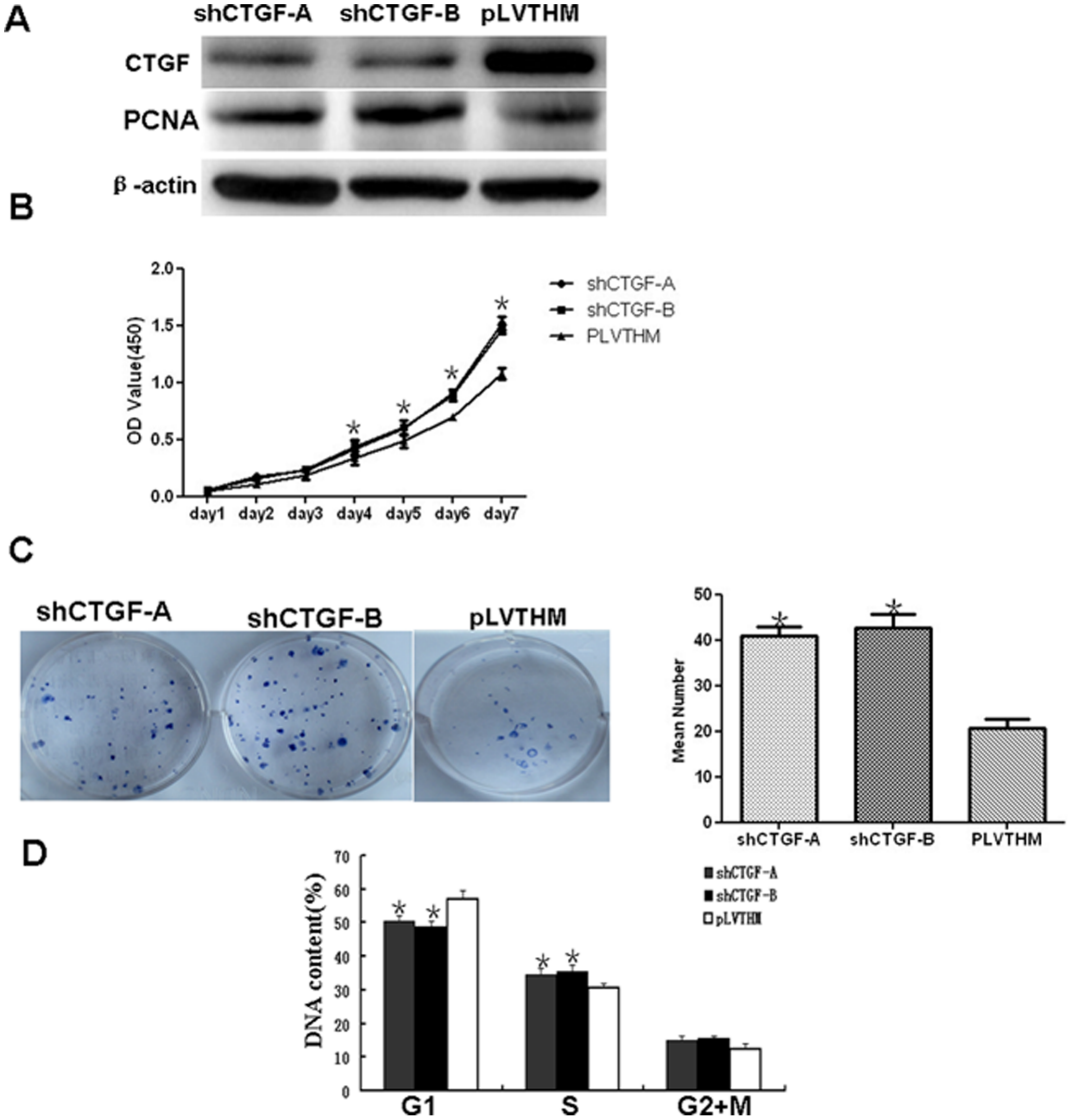
Stable suppression of CTGF expression stimulated the expression of PCNA and sped up cell proliferation, plate clone formation, and cell cycle transition from G1 to S *in vitro.* **A.** Stably knocking down CTGF increased the expression of proliferation marker PCNA in shRNA-CTGF-A and B cells compared to PLV-Ctr cells by western blot. **B.** In vitro viability of NPC cells was increased in CTGF-suppressed cells compared to PLV-Ctr cells by CCK8 assay. **C.** *In vitro* proliferative ability of NPC cells was significantly increased in CTGF-suppressed cells compared to PLV-Ctr cells by colony formation assay. **D.** Stably downregulated CTGF expression stimulated cell cycle transition from G1 to S in shRNA-CTGF-A and B cells. One-way ANOVA was used for CCK8 assay, plate clone formation and cell cycle assay. Data were presented as mean±SD for three independent experiments (*p<0.05).

**Figure 3.Transient:**
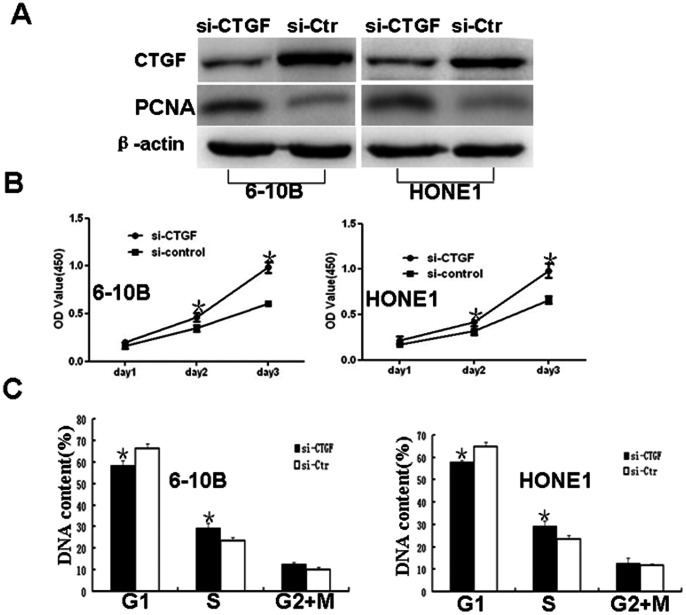
suppression of CTGF expression induced the expression of PCNA and promoted cell proliferation, plate clone formation, and cell cycle transition from G1 to S *in vitro.* **A.**Suppression of CTGF expression by siRNA induced the expression of PCNA in 6–10B cells and HONE1 cells by western blot. **B.**Transiently reducing the expression of CTGF by siRNA stimulated cell proliferation in 6–10B cells and HONE1 cells. **C.** Transiently knocking down the expression of CTGF promoted G1 to S cell cycle transition in NPC 6–10B and HONE cells. One-way ANOVA was used for CCK8 assay and cell cycle assay. Data were presented as mean±SD for three independent experiments (*p<0.05).

Among NPC cell lines, 6–10B and HONE1 cells had the highest expression levels of CTGF(Figure 1B), thus these lines were chosen to study the functions of endogenous CTGF through a loss-of-function approach. We used a lentiviral shRNA vector to specifically and stably knock down the expression of CTGF in 6–10B cell line (Figure S1). Stably decreased expression of CTGF protein induced the expression of cell proliferation marker PCNA by western blot in shRNA-1024 and 1047 cells compared to PLV-Ctr cells (Figure 2A). Subsequently, we examined the effect of decreased CTGF expression on NPC cell growth in vitro. The growth curves determined by CCK8 assay showed that suppressing CTGF significantly elevated cell viability in comparison to PLV-Ctr cells (Figure 2B). Plate clone formation assay showed that suppressing CTGF significantly elevated cell proliferation compared to PLV-Ctr cells (Figure 2C). Downregulated CTGF expression in shRNA-CTGF-1024 and 1047 cells accelerated cell cycle transition from G1 to S compared to PLV-Ctr cells (Figure 2D). Interestingly, similar results were also observed in siRNA-mediated suppression of CTGF in NPC cells. We found that knocking down endogenous CTGF expression not only elevated the expression of PCNA (Figure 3A) but also sped up cell proliferation (Figure 3B) and the G1/S transition in NPC 6–10B and HONE1 cells(Figure 3C), compared to their respective Si-Ctr-treated NPC cells. These results suggested a significant inhibitory effect of CTGF on cell growth in vitro.

### Knock-down of CTGF Facilitates Cell Migration and Invasion

**Figure 4:**
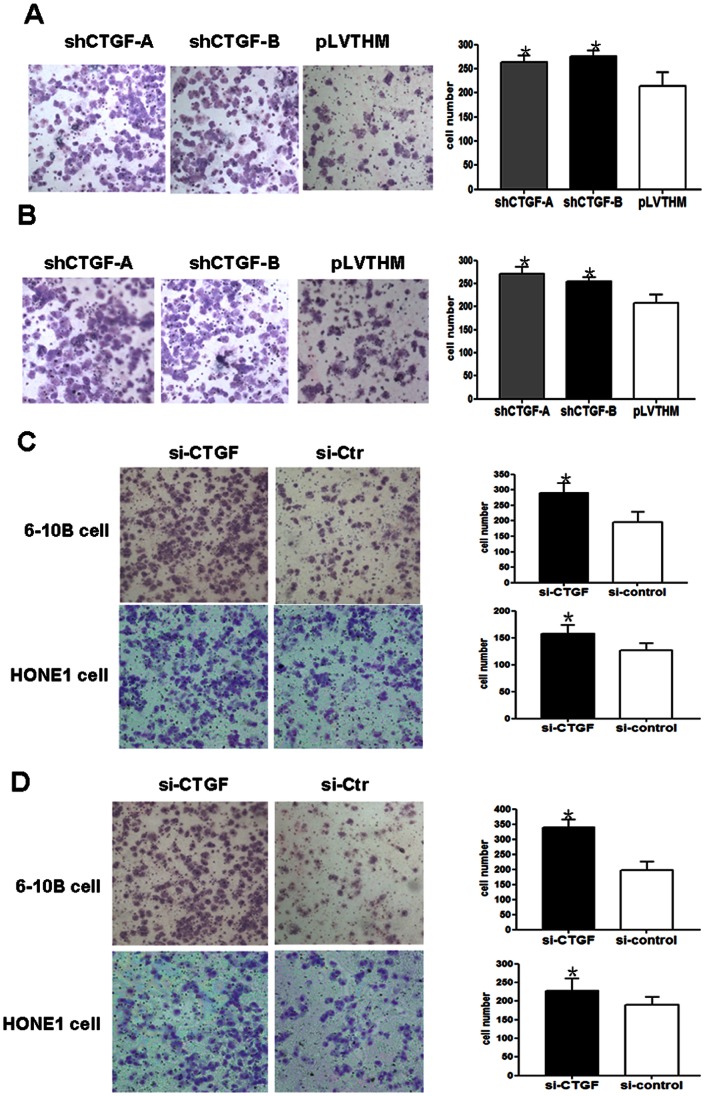
Stably or transiently inhibited CTGF expression increases cell migration and invasion. **A.** Stably downregulating CTGF enhanced the migration ability of 6–10B shRNA-CTGF-A and B cells in vitro. **B.** Stably suppressed CTGF elevated in vitro invasiveness of 6–10B shRNA-CTGF-A and B cells. **C.** Transiently downregulated CTGF dramatically enhanced the ability of 6–10B and HONE1 cells migration in vitro. **D.** Transiently suppressed CTGF elevated in vitro invasiveness of 6–10B and HONE1 cells. One-way ANOVA was used to determine the differences between two groups. (Original magnification 200×). Data were presented as mean±SD for three independent experiments (*p<0.05).

To examine the effect of CTGF on cell migration, stably shRNA-CTGF-expressing 1024 and 1047 6–10B NPC cells were cultured on transwell apparatus. After 12-h incubation, the percentage of migrated cells in both shRNA-CTGF-1024 and 1047 NPC cell groups was significantly more than that in the PLV-Ctr cells (for both P<0.001) (Figure 4A). Using a boyden chamber coated with matrigel, we determined changes in cell invasiveness after 16 h incubation. Compared with the PLV-Ctr cells, shRNA-CTGF-expressing 1024 and 1047 6–10B NPC cells both showed significantly increased invasiveness (for both P<0.001) (Figure 4B). Similar to the stably suppressed CTGF expression results, suppressing CTGF expression using siRNA-CTGF also facilitated cell migration and invasion in 6–10B and HONE1 cells (Figure 4C, D).

### CTGF Controls the Expression of Cell Cycle, MMPs, and EMT-associated Genes in NPC

**Figure 5:**
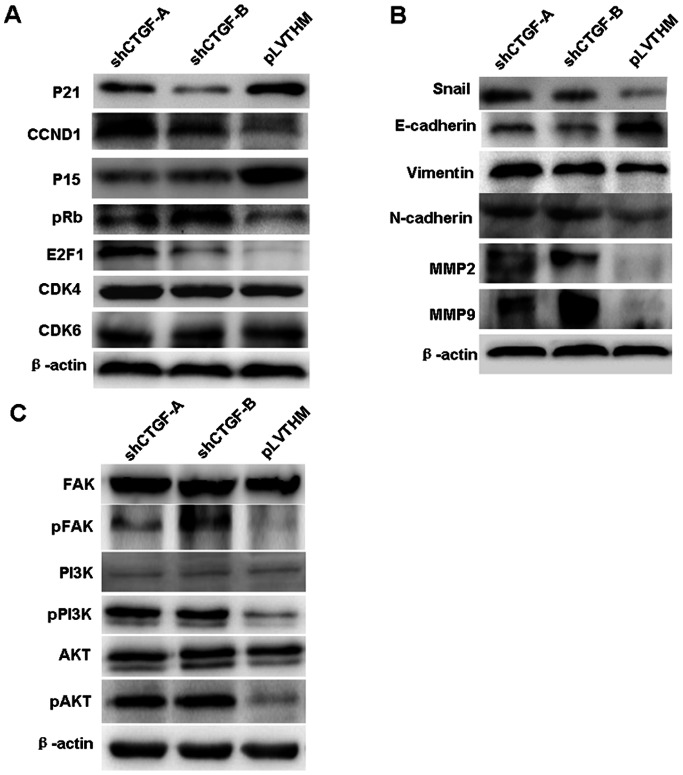
CTGF controls the expression of cell cycle, MMP, and EMT-associated genes in NPC via FAK/PI3K/AKT pathway. **A.** Knocking down endogenous CTGF expression elevated the expression of pRB(ser 780), oncogenic cell cycle regulators including CCND1, and E2F1, and suppressed the expression of tumor suppressors including p15 and p21. However, CDK4 and CDK6 were not affected. **B.** Suppressing CTGF expression increased the expression of MMP2, MMP9 and EMT-marker genes including Snail, N-cadherin, and Vimentin and decreased E-cadherin expression. **C.**Reduced CTGF expression induced the expression of phos-FAK, PI3K, and AKT, but not their total protein levels. Each experiment was repeated three times.

To further study the mechanism by which CTGF regulates cell proliferation, migration, and invasion, we examined protein levels of cell cycle, MMP, and EMT-associated genes in NPC 6–10B cells with stably suppressed CTGF expression. Knocking down endogenous CTGF expression elevated the activiation of pRB(ser 780), an oncogenic cell cycle regulator including CCND1 and E2F1, and suppressed the expression of tumor suppressors including p15 and p21. However, the expression of CDK4 and CDK6 were not affected (Figure 5A). Further, we found that suppressing CTGF expression increased the expression of MMP2, MMP9 and EMT-marker genes including Snail, N-cadherin, and Vimentin and decreased E-cadherin expression (Figure 5B). CTGF suppression did not lead to any change from epithelial to mesenchymal morphology transition in NPC 6–10B cells (Figure S2).

### CTGF Regulates FAK/PI3K/AKT Pathway

FAK/PI3K/AKT has been reported as upstream signal modulating cell cycle, EMT, and MMPs signals. We examined the effect of CTGF on FAK/PI3K/AKT pathway and found that reduced CTGF significantly increased the expression of phosphorylated FAK, PI3K and AKT, but not their total protein levels (Figure 5C). These results suggested that CTGF is an upstream factor modulating the FAK/PI3K/AKT pathway in NPC.

### CTGF Promoter Lacks Methylation in NPC

**Figure 6:**
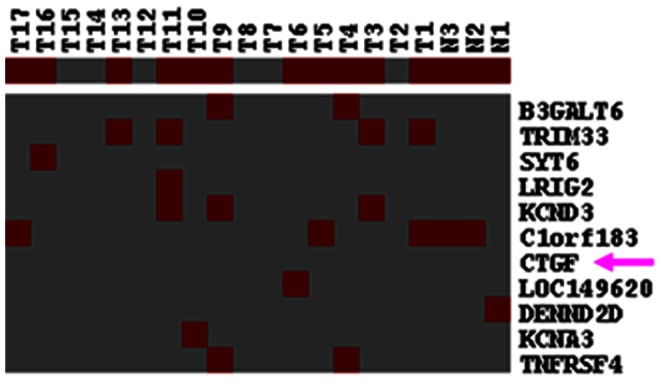
Methylation of CTGF promoter was not observed in NPC samples. Using a NimbleGen DNA methylation microarray containing CTGF, we did not detect a significant methylation change in CTGF promoter region in 17 primary NPC samples and 3 NPs.

Due to a bioinformatics-predicted CpG island in the CTGF promoter, we tested whether hypermethylation of CTGF might result in the suppressed expression of CTGF in NPC. After examination by NimbleGen DNA methylation microarray, we did not find any methylation modification in CTGF promoter region in 17 NPC samples and 3 NPs (Figure 6), which suggested that reduced expression of CTGF in NPC was not related to its promoter methylation.

## Discussion

CTGF plays dual roles as oncogene and tumor suppressor in different cancer types [6]–[14], which may be attributed to tissue-specific patterns of expression in different tissues and organs in tumourigenesis. However, its roles and molecular mechanisms linking the initiation and development of NPC are not well understood [12].

In this study, we first found that CTGF expression was decreased in NPCs compared to normal nasopharyngx (NP) tissues by microarray examination. This result strongly supported Lee et al’s microarray data (GSE2370). Further, we confirmed CTGF mRNA was weakly expressed in NPC cell lines compared to NP69 cell line or in NPC tissues compared to NPs by qPCR. These results were consistent with our microarray data, suggesting that downregulated CTGF is involved in promoting NPC pathogenesis. We used immunohistochemistry to further examine the expression level of CTGF protein in NPC tissues and noncancerous tissues. We observed that cytoplasmic CTGF expression was markedly decreased in cancer tissues compared to normal epithelium. These results were not only consistent with our previous investigation [12], but also hinted that decreased expression of CTGF was involved in the stages of NPC initiation.

In previous studies of other tumor types, different expression patterns of CTGF correlated with both favorable and unfavorable tumor progression. Elevated expression of CTGF was positively associated with progression and poor prognosis in melanoma, papillary thyroid carcinoma, esophageal squamous cell carcinoma, gastric cancer, and cervical tumors [18]–[22]. Conversely, reduced CTGF expression was favorable for tumor progression and prognosis, in oral squamous cell carcinoma, ovarian cancer, and lung adenocarcinomas [23]–[25]. In this study, we found that attenuated CTGF expression was negatively associated with T, N classification, and clinical stages of NPC patients. The results suggested the downregulated expression of CTGF promoted NPC pathogenesis. To specifically determine the contributions of CTGF in the regulation of NPC phenotypes, we modulated its expression in 6–10B cell lines. We found that stably decreased expression of CTGF by shRNA conferred 6–10B cells with higher expression of proliferation marker protein PCNA, cell proliferation, colony formation, G1/S cell cycle transition, migration and invasion *in vitro*. Similar results were observed after transiently suppressing CTGF expression by siRNA transfection in NPC 6–10B and HONE1 cells.

The biological functions of CTGF found in this study provided a mechanistic basis for the pathological and clinical observations. We examined key cell cycle regulators of the G1-S transition and observed that CCND1, pRb, and E2F1 were upregulated while p15 and p21 were downregulated after stable CTGF knockdown in 6–10B cells. Further, we found that CTGF suppression-induced expression of genes is associated with cell migration and invasion. MMP2, MMP9, and EMT-marker genes including Snail, N-cadherin, and Vimentin were highly upregulated while EMT-marked gene E-cadherin was weakly expressed in shRNA treated 6–10B cells. However, CTGF suppression did not lead to any change from epithelial to mesenchymal morphology changes in NPC 6–10B cells.

PI3K/AKT is a classical signal pathway [26], [27] and its activated status induces ell cycle transition of G1/S [28], increases the expression of Snail promoting the EMT [29], [30] and stimulates the secretion of MMP2 and MMP9 [31]. This signaling respectively promotes cell proliferation, migration, and invasion during tumor pathogenesis. In previous investigation of oral cancer, CTGF was reported to inhibit cell motility and COX-2 expression through the FAK/PI3K/AKT pathway [15]. We conjectured that decreased CTGF expression promoted cell growth, migration, and invasion via the same pathway activity in NPC. In this study, we also observed that decreased CTGF expression increased pFAK, pPI3K, and pAKT levels, while not afftecting total FAK, PI3K, and AKT protein levels. Furthermore, we also observed that inhibiting PI3K expression downregulated the expression of PI3K, pPI3K, and pAKT. However, a change in CTGF expression was not observed. These results demonstrated that attenuated CTGF expression is an upstream factor involved in activation of the FAK/PI3K/AKT pathway in NPC.

The hypermethylation of CpG islands in gene promoters can often lead to transcriptional silencing of genes, including tumor suppressor genes. Due to the existence of predicted CpG islands and hypermethylation of CTGF promoter region in ovarian cancers [24], we used a NimbleGen DNA methylation microarray to assess its methylation status in 17 NPC cases. However, there were no significant changes in CTGF promoter methylation observed in these samples, suggesting the involvement of other mechanisms in suppressing CTGF expression in NPC.

In summary, this study provides evidence that CTGF is downregulated in NPC and its reduced cytoplasmic expression facilitates disease progression. Reduced CTGF levels lead to elevated cell proliferation, migration, invasion, and cell cycle progression by activating the FAK/PI3K/AKT pathway. Our studies demonstrated that CTGF plays a potential tumor suppressor role in NPC pathogenesis.

## Supporting Information

Figure S1 **The efficiency of infection was determined by the numbers of cells with green fluorescent protein (GFP) which were infected by viruses labeled with GFP.** Cells are presented at 100 times magnification.
(TIF)

Figure S2 **Stably knocking down the CTGF expression did not lead to epithelial to mesenchymal transition morphology changes in NPC 6–10B cells.**
(TIF)
