# Supplementary figures and images for: Reduced CTGF Expression Promotes Cell Growth, Migration, and Invasion in Nasopharyngeal Carcinoma
Source: PLoS One. 2013 Jun 3;8(6):e64976. doi: 10.1371/journal.pone.0064976 (PMC3670884; doi:10.1371/journal.pone.0064976)

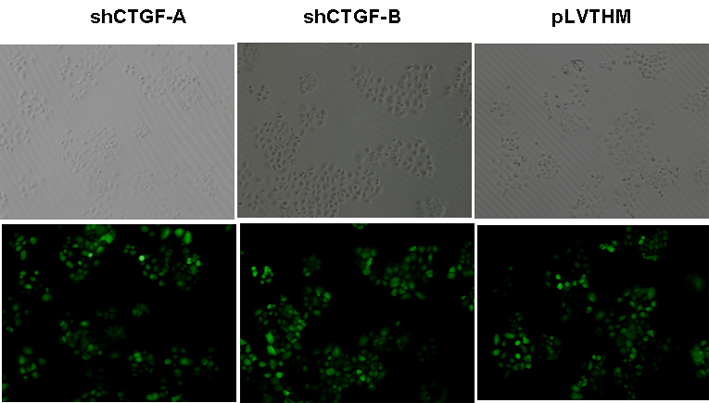

Supplement: Figure S1 — The efficiency of infection was determined by the numbers of cells with green fluorescent protein (GFP) which were infected by viruses labeled with GFP. Cells are presented at 100 times magnification. (TIF) [file pone.0064976.s001.tif]

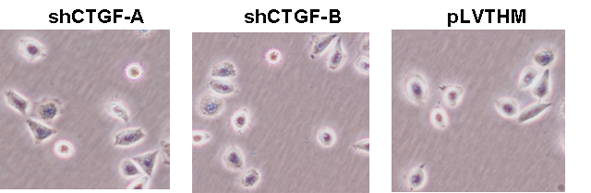

Supplement: Figure S2 — Stably knocking down the CTGF expression did not lead to epithelial to mesenchymal transition morphology changes in NPC 6–10B cells. (TIF) [file pone.0064976.s002.tif]
