# Supplementary material for: Expression of Concern: Reduced CTGF expression promotes cell growth, migration, and invasion in nasopharyngeal carcinoma
Source: PLoS One. 2020 Apr 2;15(4):e0231520. doi: 10.1371/journal.pone.0231520 (PMC7117724; doi:10.1371/journal.pone.0231520)
Supplement: S3 File — (DOC) [file pone.0231520.s003.doc]

1. **p21,CCND1,P15,CDK4,CDK6,N-Ca,MMP2,MMP9,FAK and pFAK( original pictures in paper)**

**p21**


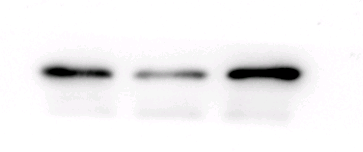


shCTGF-A shCTGF-B pLVTHM

**CCND1**

shCTGF-A shCTGF-B pLVTHM


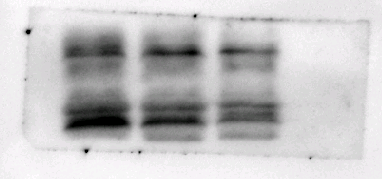


**P15**


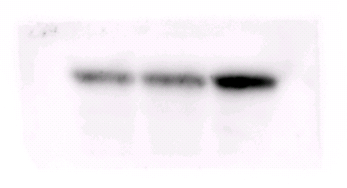


shCTGF-A shCTGF-B pLVTHM

**CDK4**


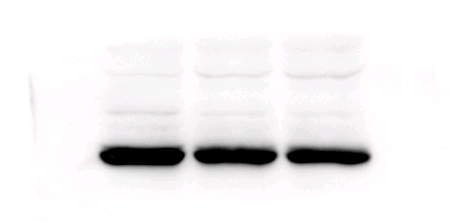


shCTGF-A shCTGF-B pLVTHM

**CDK6**
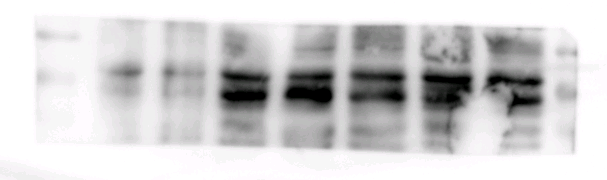


shCTGF-A shCTGF-B pLVTHM

**N-Ca**
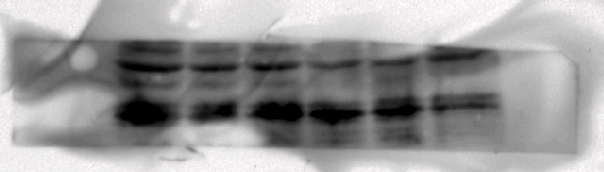


shCTGF-A shCTGF-B pLVTHM

**MMP2**


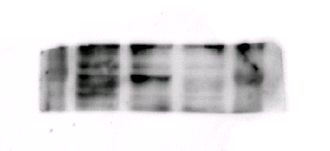


shCTGF-A shCTGF-B pLVTHM

**MMP9**


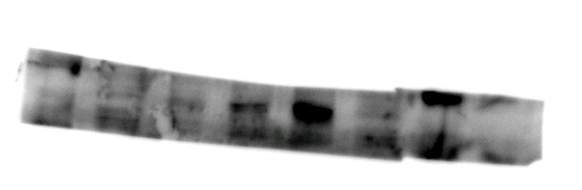


shCTGF-A shCTGF-B pLVTHM

**FAK**


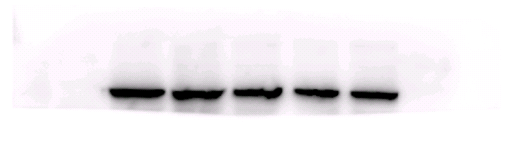


shCTGF-A shCTGF-B pLVTHM

**pFAK**


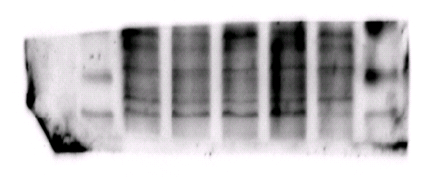


shCTGF-A shCTGF-B pLVTHM

1. **CTGF，β-actin，PI3K, pPI3K, pAKT (original pictures from different replicates published in master thesis)**

**CTGF**(Fig.2.4,Page 31 of masters thesis)

pLVTHM shCTGF-A shCTGF-B


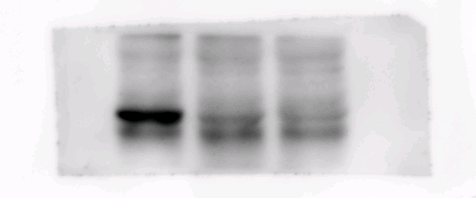


**β-actin**(Fig.2.4,Page 31 of masters thesis)

**
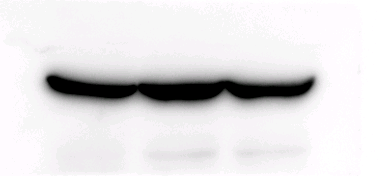
**

pLVTHM shCTGF-A shCTGF-B

**β-actin**（Fig.4.1,Page 70 of masters thesis）


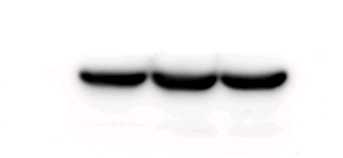


pLVTHM shCTGF-A shCTGF-B

**PI3K**（Fig.4.1,Page 70 of masters thesis）


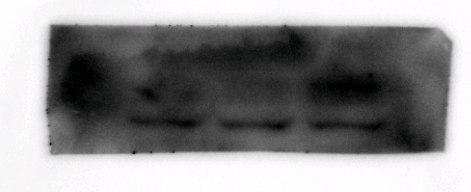


pLVTHM shCTGF-A shCTGF-B

**pPI3K**（Fig.4.1,Page 71 of masters thesis）


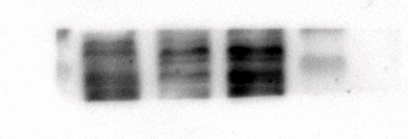


pLVTHM shCTGF-A shCTGF-B

**pAKT**（Fig.4.1,Page 71 of masters thesis）


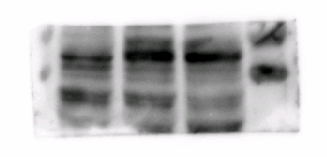


pLVTHM shCTGF-A shCTGF-B
