# Supplementary material for: Correction to Expression of Concern on: Reduced CTGF expression promotes cell growth, migration, and invasion in nasopharyngeal carcinoma
Source: PLoS One. 2020 May 21;15(5):e0233787. doi: 10.1371/journal.pone.0233787 (PMC7241696; doi:10.1371/journal.pone.0233787)
Supplement: S1 File — (PDF) [file pone.0233787.s001.pdf]

PLOS ONE

EXPRESSION OF CONCERN

Expression of concern: Reduced CTGF expression promotes cell growth, migration, and invasion in nasopharyngeal carcinoma

The *PLOS ONE* Editors

After publication of this article [1], concerns were raised about the identity of the cell lines used in the study, regions of overlap between some image panels in Fig 4, western blot methodology and representation in Fig 5, and the availability of the underlying data.

Four of the eight nasopharyngeal carcinoma (NPC) cell lines used in the qPCR experiment shown in Fig 1B have been reported elsewhere to be possibly contaminated or misidentified (CNE1, CNE2, HONE1, and HNE1, see ICLAC Register of Misidentified Cell Lines (iclac.org/databases/cross-contaminations/) [2], Cellosaurus (https://web.expasy.org/cellosaurus/) [3] and [4–5]). Of these potentially misidentified cell lines, only HONE1 was used in the further functional studies reported in the article. The corresponding author has provided STR profile reports for the 5-8F cells and the HONE1 cells (S1 File).

In addition to HONE1, the follow-up experiments reported in [1] use only one other NPC cell line, 6-10B, which may limit the generalizability of the findings as representative of nasopharyngeal cancer biology.

Clarification of the source and provenance of the cell lines used is as follows:

- 5-8F, 6-10B, and SUNE1 are from stock kept at the Cancer Institute of Southern Medical University since 2003, originally obtained from Cancer Center, Sun Yat-sen University where these cell lines were generated [6, 7].
- C666-1 cell line was generated by the Chinese University of Hong Kong [8]. It is from stock kept by the Cancer Institute of Southern Medical University since 2003, originally obtained from Cancer Center of Sun Yat-sen University.
- CNE1 and CNE2 are from stock kept by the Cancer Institute of Southern Medical University since 1999.
- HONE1 and HNE1 are from stock kept by the Cancer Institute of Southern Medical University, originally obtained from the Cancer Institute of Hunan Medical University (now the Central Southern University) where these cell lines were generated.
- NP69 [9] is from stock kept by the Cancer Institute of Southern Medical University, since 2009, originally obtained from Cancer Center, Sun Yat-sen University where the cell line was generated.

In Fig 4C, it was noted that the si-CTGF panel in 6-10B cells is the same as Fig 4D si-CTGF panel in 6-10B cells. Additionally, there is a region of overlap between the images in Fig 4C si-CTGF panel in HONE1 cells and Fig 4C si-Ctr in HONE1 cells. The authors confirmed that incorrect image files were used for the following panels:

- Fig 4A shCTGF-B panel;

Check for updates

OPEN ACCESS

Citation: The *PLOS ONE* Editors (2020) Expression of concern: Reduced CTGF expression promotes cell growth, migration, and invasion in nasopharyngeal carcinoma. PLoS ONE 15(4): e0231520. https://doi.org/10.1371/journal.pone.0231520

Published: April 2, 2020

Copyright: © 2020 The PLOS ONE Editors. This is an open access article distributed under the terms of the Creative Commons Attribution License, which permits unrestricted use, distribution, and reproduction in any medium, provided the original author and source are credited.

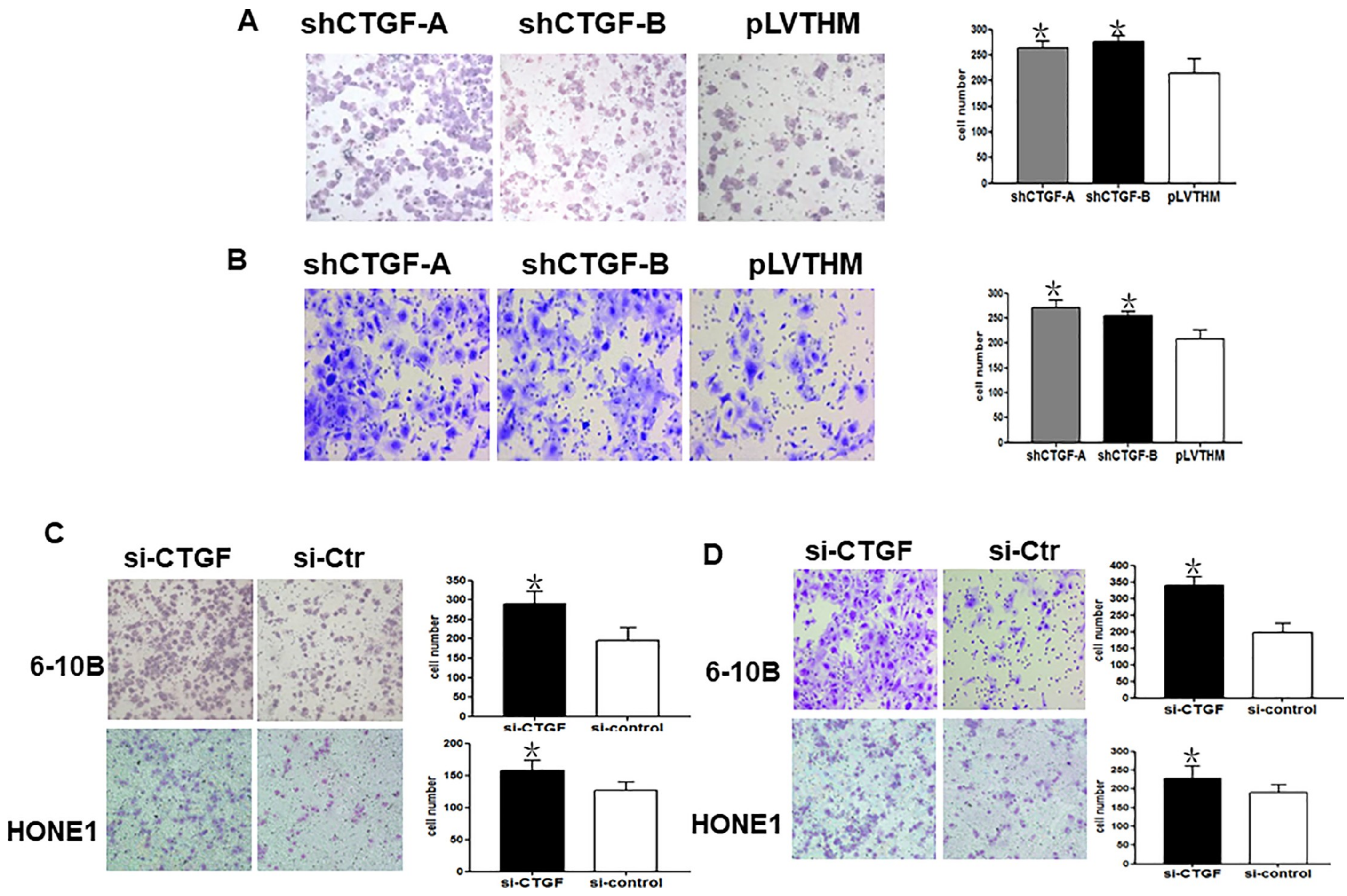

Fig 1. Reduced expression of CTGF promotes the progression and poor prognosis of NPC patients. A. Decreased CTGF expression was shown in NPC cells compared to nasopharynx tissues by microarray data analysis of GSE2370 set retrieved from the GEO database (Red color: high expression,Green color:low expression). **B.** Compared with immortalized human nasopharyngeal epithelial NP69 cell line, CTGF expression was significantly downregulated in 8 NPC cell lines. **C.** Compared with 11 NP tissues, CTGF expression was markedly decreased in 20 primary NPC tissues (p = 0.0165). The unpaired t test was used for this assay (*p<0.05). **D.** CTGF expression in primary NPC samples and NP tissues 1,2)High expression in nasopharyngeal epithelium; 3,4)High expression in NPC; 5,6): Low expression in NPC (Original magnification 400×).

https://doi.org/10.1371/journal.pone.0231520.g002

- All panels of Fig 4B;
- Fig 4C HONE1 cell, si-CTGF panel;
- Fig 4C HONE1 cell, si-Ctr panel;
- All panels of Fig 4D.

A revised Fig 4 is provided here in which the incorrect image panels are replaced. The underlying data for Fig 4, including the representative image files, other replicate image files, and individual-level datasets for the charts, are provided as S2 File. The authors stated that the errors in selecting the representative image files for Fig 4 do not affect the statistical data collected and reported in the accompanying charts.

In Fig 5, the authors clarified that the β-actin panels shown are representative only, and do not show the actual loading controls for all target proteins shown in parts A, B and C, respectively. Actin loading controls were not run on the same gels as target proteins in each

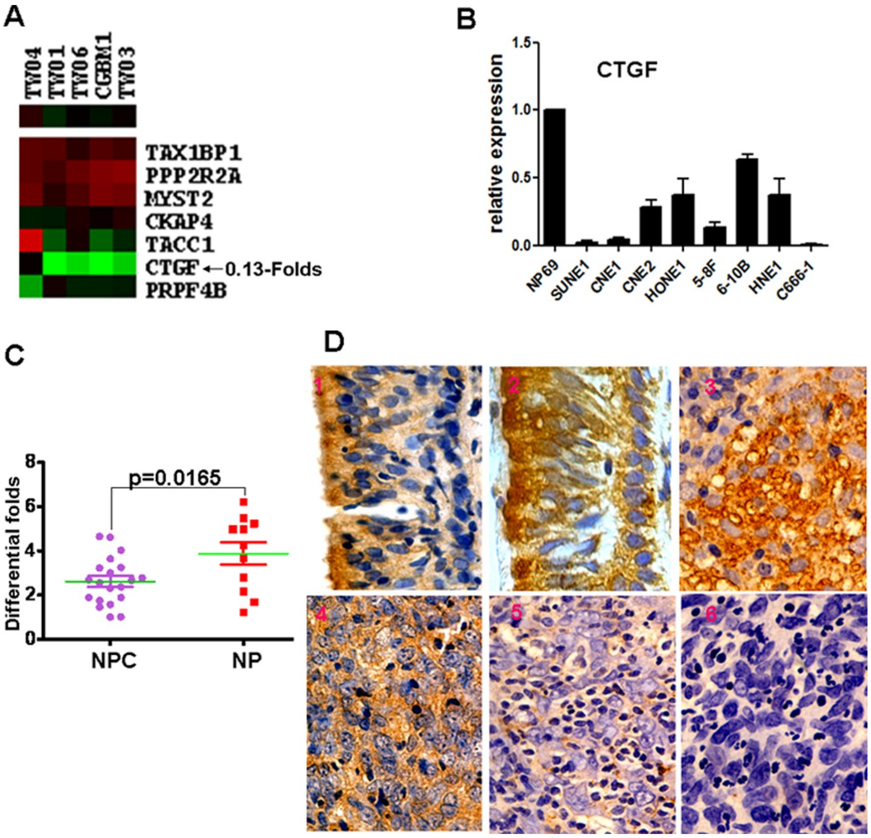

Fig 4. Stably or transiently inhibited CTGF expression increases cell migration and invasion. **A.** Stably downregulating CTGF enhanced the migration ability of 6–10B shRNA-CTGF-A and B cells in vitro. **B.** Stably suppressed CTGF elevated in vitro invasiveness of 6–10B shRNA-CTGF-A and B cells. **C.** Transiently downregulated CTGF dramatically enhanced the ability of 6–10B and HONE1 cells migration in vitro. **D.** Transiently suppressed CTGF elevated in vitro invasiveness of 6–10B and HONE1 cells. One-way ANOVA was used to determine the differences between two groups. (Original magnification 200×). Data were presented as mean±SD for three independent experiments ($^{*}p<0.05$).

https://doi.org/10.1371/journal.pone.0231520.g001

experiment. Protein quantification was carried out to ensure equal sample loading, and blots were checked for consistency of actin control bands. Target protein expression levels were compared when actin protein expression was observed to be relatively consistent. The available uncropped western blot images underlying Fig 5 are provided as Supporting Information (S3 File).

The underlying data files are no longer available for the immunohistochemistry images in Fig 1D, the western blots in Figs 2A and 3A, the western blots for PI3K, pPI3K, AKT, pAKT, pRb, E2F1, Snail, E-cadherin, Vimentin and β-actin in Fig 5, and the charts in Figs 2B and 3B.

The raw data for the NimbleGen DNA methylation microarray experiment reported in Fig 6 were not deposited in a public repository as required by the standards of the field. The dataset was subsequently deposited and is available at https://datadryad.org/stash/dataset/doi:10.5061/dryad.wh70rxwj1. No additional underlying data are available for this article.

The following additional issues were noted:

The Transient Transfection with siRNAs subsection of the Materials and Methods of [1] states that the siRNA sequences for the gene and the controls are provided in Table 3. However, the control sequences are not provided. The control siRNA sequences were designed and synthesized by Guangzhou RiboBio (RiboBio Inc, China) and are protected under a patent.

The antisense strand of the siRNAs targeting CTGF are:

5' UAUGUCUUCAUGCUGGUGC dTdT 3'

5' UAAUCAUAGUUGGGUCUGG dTdT 3'

5' UUUGGGAGUACGGAUGCAC dTdT 3'

These sequences are reported in Table 2 of [1] in 3'-5' direction.

There is an error in the text legend for Fig 1D. A complete, correct Fig 1 legend is provided here.

The *PLOS ONE* Editors issue this Expression of Concern to alert readers to the concerns about the accuracy of the representation of the western blot data and of the migration and invasion image panels, and the use of cell lines previously reported to be misidentified.

Supporting information

S1 File. STR profile reports. STR profiles of HONE1 and 5-8F cell line samples, analysed in Dec 2017 and Jan 2018, respectively; search results in ATCC and DSMZ databases; and electrophoresis of gene COX1.
(ZIP)

S2 File. The underlying data for Fig 4. Representative image files, other replicate image files, and individual-level datasets for the charts.
(RAR)

S3 File. The available uncropped western blot images underlying Fig 5.
(DOC)

References

1. Zhen Y, Ye Y, Yu X, Mai C, Zhou Y, Chen Y, et al. (2013) Reduced CTGF Expression Promotes Cell Growth, Migration, and Invasion in Nasopharyngeal Carcinoma. PLoS ONE 8(6): e64976. https://doi.org/10.1371/journal.pone.0064976 PMID: 23755163
2. Capes-Davis A, Theodosopoulos G, Atkin I, Drexler HG, Kohara A, MacLeod RA, et al. Check your cultures! A list of cross-contaminated or misidentified cell lines. Int J Cancer. 2010; 127(1):1–8. https://doi.org/10.1002/ijc.25242 PMID: 20143388
3. Bairoch A. The Cellosaurus, a Cell-Line Knowledge Resource. J Biomol Tech. 2018; 29(2):25–38. https://doi.org/10.7171/jbt.18-2902-002 PMID: 29805321
4. Chan SY-Y, Choy K-W, Tsao S-W, Tao Q, Tang T, Chung GT-Y and Lo K-W (2008), Authentication of nasopharyngeal carcinoma tumor lines. Int. J. Cancer, 122: 2169–2171. https://doi.org/10.1002/ijc.23374 PMID: 18196576
5. Strong MJ, Baddoo M, Nanbo A, Xu M, Puetter A, Lin Z. Comprehensive High-Throughput RNA Sequencing Analysis Reveals Contamination of Multiple Nasopharyngeal Carcinoma Cell Lines with HeLa Cell Genomes. J Virol. 2014; 88 (18) 10696–10704; https://doi.org/10.1128/JVI.01457-14 PMID: 24991015
6. Song LB, Yan J, Jian SW, Zhang L, Li MZ, Li D, Wang HM.Molecular mechanisms of tumorgenesis and metastasis in nasopharyngeal carcinoma cell sublines. Ai Zheng. 2002 Feb; 21(2):158–62. Chinese. PMID: 12479066
7. Zhang L, Song L, Ma Y, Huang B, Liang Q, Zeng Y.Differentially expressed gene in nasopharyngeal carcinoma cell lines with various metastatic potentialities. Zhonghua Zhong Liu Za Zhi. 2002 Sep; 24 (5):430–4. Chinese. PMID: 12485491
8. Cheung ST, Huang DP, Hui AB, Lo KW, Ko CW, Tsang YS, et al. Nasopharyngeal carcinoma cell line (C666-1) consistently harbouring Epstein-Barr virus. Int J Cancer. 1999 Sep 24; 83(1):121–6. https://doi.org/10.1002/(sici)1097-0215(19990924)83:1<121::aid-ijc21>3.0.co;2-f PMID: 10449618
9. Song LB, Zeng MS, Liao WT, Zhang L, Mo HY, Liu WL et al. Bmi-1 is a novel molecular marker of nasopharyngeal carcinoma progression and immortalizes primary human nasopharyngeal epithelial cells. Cancer Res. 2006; 66:6225–6232 https://doi.org/10.1158/0008-5472.CAN-06-0094 PMID: 16778197
